# (*R*)-(3-Carb­oxy-2-hy­droxy­prop­yl)tri­methyl­aza­nium chloride

**DOI:** 10.1107/S160053681201598X

**Published:** 2012-04-18

**Authors:** Seik Weng Ng

**Affiliations:** a Department of Chemistry, University of Malaya, 50603 Kuala Lumpur, Malaysia, and Chemistry Department, Faculty of Science, King Abdulaziz University, PO Box 80203 Jeddah, Saudi Arabia

## Abstract

In the title salt (common name l-carnitine hydro­chloride), C_7_H_16_NO_3_^+^·Cl^−^, the organic cation features a carb­oxy­lic part (–CO_2_H) having unambigous single- and double-bonds [1.336 (2), 1.211 (2) Å]. There is a large N—C—C bond angle [115.9 (1)°] for the C atom connected to the bulky trimethyl­amino substituent. In the crystal, the acid H atom forms a hydrogen bond to the chloride anion, whereas the hydroxyl H atom forms a longer hydrogen bond to the anion, generating a helical chain running along [001].

## Related literature

For racemic carnitine hydro­chloride, see: Tomita *et al.* (1974 ▶); Yunuskhodzhaev *et al.* (1991 ▶). For *R*-carnitine, see: Gandour *et al.* (1985 ▶).
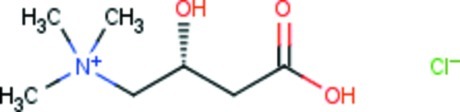


## Experimental

### 

#### Crystal data

C_7_H_16_NO_3_^+^·Cl^−^
*M* *_r_* = 197.66
Orthorhombic, 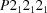

*a* = 6.3043 (3) Å
*b* = 11.5256 (7) Å
*c* = 13.4905 (8) Å
*V* = 980.23 (10) Å^3^
*Z* = 4
Mo *K*α radiation
μ = 0.36 mm^−1^
*T* = 100 K
0.40 × 0.30 × 0.20 mm

#### Data collection

Agilent Technologies SuperNova Dual diffractometer with Atlas detector
Absorption correction: multi-scan *CrysAlis PRO* (Agilent, 2012 ▶) *T*_min_ = 0.869, *T*_max_ = 0.931
6682 measured reflections
2251 independent reflections
2191 reflections with *I* > 2σ(*I*)
*R*_int_ = 0.033

#### Refinement

*R*[*F* ^2^ > 2σ(*F* ^2^)] = 0.027
*wR*(*F* ^2^) = 0.072
*S* = 1.09
2251 reflections
117 parameters
2 restraints
H atoms treated by a mixture of independent and constrained refinement
Δρ_max_ = 0.28 e Å^−3^
Δρ_min_ = −0.19 e Å^−3^
Absolute structure: Flack (1983 ▶), 926 Friedel pairs
Flack parameter: 0.01 (5)

### 

Data collection: *CrysAlis PRO* (Agilent, 2012 ▶); cell refinement: *CrysAlis PRO*; data reduction: *CrysAlis PRO*; program(s) used to solve structure: *SHELXS97* (Sheldrick, 2008 ▶); program(s) used to refine structure: *SHELXL97* (Sheldrick, 2008 ▶); molecular graphics: *X-SEED* (Barbour, 2001 ▶); software used to prepare material for publication: *publCIF* (Westrip, 2010 ▶).

## Supplementary Material

Crystal structure: contains datablock(s) global, I. DOI: 10.1107/S160053681201598X/xu5512sup1.cif

Structure factors: contains datablock(s) I. DOI: 10.1107/S160053681201598X/xu5512Isup2.hkl

Supplementary material file. DOI: 10.1107/S160053681201598X/xu5512Isup3.cml

Additional supplementary materials:  crystallographic information; 3D view; checkCIF report

## Figures and Tables

**Table 1:** Hydrogen-bond geometry (Å, °)

*D*—H⋯*A*	*D*—H	H⋯*A*	*D*⋯*A*	*D*—H⋯*A*
O1—H1⋯Cl1	0.85 (1)	2.18 (1)	3.022 (1)	176 (2)
O3—H3⋯Cl1^i^	0.83 (1)	2.51 (2)	3.209 (1)	142 (2)

Symmetry code: (i) 
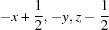
.

**Fig. 1.**
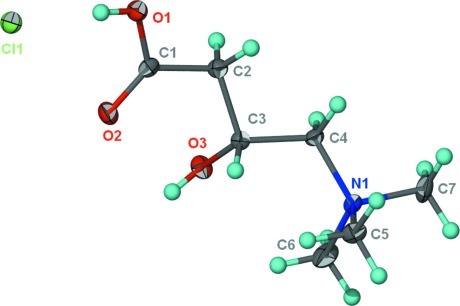
Thermal ellipsoid plot (Barbour, 2001) of C7H16NO3+.Cl- at the 70% probability level; hydrogen atoms are drawn as spheres of arbitrary radius.

## supplementary crystallographic information

### Comment

A model for the binding of *L*-carnitine to the carnitine acetyltransferase enzyme has been proposed on the basis of the crystal structure of *L*-carnitine (Gandour *et al.*, 1985). *L*-Carnitine, a zwitterionic compound that is biosynthesized from lysine and methinonine, is the vitamin B_T_; it is also avaliable commerically as the hydrochloride salt. The crystal structure of racemic carnitine hydrochloride has been previously reported (Tomita *et al.*, 1974; Yunuskhodzhaev *et al.*, 1991). In the crystal structure of *L*-carnitine hydrochloride (Scheme I), the carboxyl –CO_2_ part carries the acid hydrogen (Fig. 1). This part has unambigous single- and double-bonds [1.336 (2), 1.211 (2) Å]. The three-atom C_carboxyl_–*C*–C_trimethylamino_ unit shows a large angle [115.9 (1) °] for the atom connected to the bulky trimethylamino substituent. The acid hydrogen forms a hydrogen bond to the chloride anion (Table 1). Oddly, the hydroxy group does not engage in any hydrogen bonding interactions.

### Experimental

*L*-Carnithine hydrochloride as supplied by Sigma Chemical Company consists of colorless prismatic crystals, and was used without purification.

### Refinement

Carbon-bound H-atoms were placed in calculated positions [C–H 0.98 to 1.00 Å, *U*_iso_(H) 1.2 to 1.5*U*_eq_(C)] and were included in the refinement in the riding model approximation.

The hydroxy and acid H-atoms were located in a difference Fourier map, and were refined with a distance restraint of O–H 0.84±0.01 Å; their temperature factors were refined.

### Crystal data

**Table:**

C_7_H_16_NO_3_^+^·Cl^−^	*F*(000) = 424
*M**_r_* = 197.66	*D*_x_ = 1.339 Mg m^−^^3^
Orthorhombic, *P*2_1_2_1_2_1_	Mo *K*α radiation, λ = 0.71073 Å
Hall symbol: P 2ac 2ab	Cell parameters from 3837 reflections
*a* = 6.3043 (3) Å	θ = 2.3–27.5°
*b* = 11.5256 (7) Å	µ = 0.36 mm^−^^1^
*c* = 13.4905 (8) Å	*T* = 100 K
*V* = 980.23 (10) Å^3^	Prism, colorless
*Z* = 4	0.40 × 0.30 × 0.20 mm

### Data collection

**Table:**

Agilent Technologies SuperNova Dual diffractometer with Atlas detector	2251 independent reflections
Radiation source: SuperNova (Mo) X-ray Source	2191 reflections with *I* > 2σ(*I*)
Mirror monochromator	*R*_int_ = 0.033
Detector resolution: 10.4041 pixels mm^-1^	θ_max_ = 27.5°, θ_min_ = 2.3°
ω scan	*h* = −8→8
Absorption correction: multi-scan *CrysAlis PRO* (Agilent, 2012)	*k* = −12→15
*T*_min_ = 0.869, *T*_max_ = 0.931	*l* = −16→17
6682 measured reflections	

### Refinement

**Table:**

Refinement on *F*^2^	Secondary atom site location: difference Fourier map
Least-squares matrix: full	Hydrogen site location: inferred from neighbouring sites
*R*[*F*^2^ > 2σ(*F*^2^)] = 0.027	H atoms treated by a mixture of independent and constrained refinement
*wR*(*F*^2^) = 0.072	*w* = 1/[σ^2^(*F*_o_^2^) + (0.0381*P*)^2^ + 0.129*P*] where *P* = (*F*_o_^2^ + 2*F*_c_^2^)/3
*S* = 1.09	(Δ/σ)_max_ = 0.001
2251 reflections	Δρ_max_ = 0.28 e Å^−^^3^
117 parameters	Δρ_min_ = −0.19 e Å^−^^3^
2 restraints	Absolute structure: Flack (1983), 926 Friedel pairs
Primary atom site location: structure-invariant direct methods	Flack parameter: 0.01 (5)

### Fractional atomic coordinates and isotropic or equivalent isotropic displacement parameters (Å^2^)

**Table:**

	*x*	*y*	*z*	*U*_iso_*/*U*_eq_	
Cl1	0.16829 (6)	−0.02923 (3)	1.11989 (3)	0.01599 (10)	
O1	0.11377 (16)	0.20168 (9)	1.01683 (8)	0.0153 (2)	
H1	0.123 (3)	0.1372 (11)	1.0468 (14)	0.032 (6)*	
O2	0.09374 (17)	0.09692 (9)	0.87751 (9)	0.0164 (2)	
O3	0.03544 (17)	0.23605 (10)	0.69856 (8)	0.0146 (2)	
H3	0.066 (4)	0.1658 (10)	0.6948 (18)	0.047 (7)*	
N1	0.38932 (18)	0.40990 (10)	0.62406 (10)	0.0107 (2)	
C1	0.1083 (2)	0.19013 (12)	0.91830 (11)	0.0113 (3)	
C2	0.1258 (2)	0.30406 (12)	0.86490 (11)	0.0120 (3)	
H2A	0.2287	0.3542	0.9001	0.014*	
H2B	−0.0138	0.3435	0.8659	0.014*	
C3	0.1976 (2)	0.28843 (12)	0.75711 (10)	0.0103 (3)	
H3A	0.3279	0.2390	0.7552	0.012*	
C4	0.2485 (2)	0.40787 (13)	0.71538 (11)	0.0109 (3)	
H4A	0.1132	0.4470	0.6989	0.013*	
H4B	0.3182	0.4540	0.7680	0.013*	
C5	0.6024 (2)	0.35642 (13)	0.64467 (12)	0.0151 (3)	
H5A	0.6887	0.3582	0.5843	0.023*	
H5B	0.5834	0.2758	0.6661	0.023*	
H5C	0.6741	0.4003	0.6971	0.023*	
C6	0.2902 (3)	0.35091 (15)	0.53684 (11)	0.0198 (4)	
H6A	0.3867	0.3552	0.4800	0.030*	
H6B	0.1563	0.3895	0.5203	0.030*	
H6C	0.2628	0.2694	0.5531	0.030*	
C7	0.4241 (3)	0.53502 (13)	0.59814 (12)	0.0187 (3)	
H7A	0.5126	0.5402	0.5386	0.028*	
H7B	0.4954	0.5742	0.6533	0.028*	
H7C	0.2871	0.5723	0.5856	0.028*	

### Atomic displacement parameters (Å^2^)

**Table:**

	*U*^11^	*U*^22^	*U*^33^	*U*^12^	*U*^13^	*U*^23^
Cl1	0.02265 (18)	0.01262 (16)	0.01271 (18)	0.00108 (14)	0.00145 (14)	0.00228 (14)
O1	0.0237 (6)	0.0118 (5)	0.0104 (5)	0.0005 (5)	0.0008 (4)	0.0021 (4)
O2	0.0218 (5)	0.0124 (5)	0.0150 (5)	−0.0037 (4)	−0.0005 (5)	−0.0013 (4)
O3	0.0161 (5)	0.0142 (5)	0.0135 (6)	−0.0021 (5)	−0.0049 (4)	−0.0009 (4)
N1	0.0103 (6)	0.0117 (5)	0.0102 (6)	0.0002 (5)	0.0010 (5)	0.0014 (5)
C1	0.0073 (6)	0.0149 (7)	0.0118 (7)	0.0007 (6)	0.0006 (5)	0.0008 (6)
C2	0.0126 (7)	0.0121 (6)	0.0112 (7)	0.0013 (6)	0.0002 (5)	0.0003 (5)
C3	0.0095 (6)	0.0109 (6)	0.0106 (7)	0.0008 (5)	−0.0011 (5)	0.0004 (6)
C4	0.0114 (6)	0.0115 (6)	0.0099 (7)	0.0010 (6)	0.0024 (5)	0.0004 (6)
C5	0.0118 (7)	0.0180 (7)	0.0155 (8)	0.0044 (6)	0.0011 (6)	0.0008 (6)
C6	0.0210 (8)	0.0305 (9)	0.0081 (8)	−0.0080 (7)	−0.0022 (6)	−0.0002 (6)
C7	0.0167 (7)	0.0134 (7)	0.0260 (9)	−0.0003 (7)	0.0062 (6)	0.0078 (7)

### Geometric parameters (Å, º)

**Table:**

O1—C1	1.3363 (18)	C3—C4	1.521 (2)
O1—H1	0.848 (9)	C3—H3A	1.0000
O2—C1	1.2105 (18)	C4—H4A	0.9900
O3—C3	1.4262 (18)	C4—H4B	0.9900
O3—H3	0.833 (9)	C5—H5A	0.9800
N1—C6	1.4956 (19)	C5—H5B	0.9800
N1—C7	1.5000 (19)	C5—H5C	0.9800
N1—C5	1.5042 (18)	C6—H6A	0.9800
N1—C4	1.5188 (18)	C6—H6B	0.9800
C1—C2	1.5018 (19)	C6—H6C	0.9800
C2—C3	1.534 (2)	C7—H7A	0.9800
C2—H2A	0.9900	C7—H7B	0.9800
C2—H2B	0.9900	C7—H7C	0.9800
			
C1—O1—H1	112.9 (14)	N1—C4—H4A	108.3
C3—O3—H3	106.3 (18)	C3—C4—H4A	108.3
C6—N1—C7	108.35 (12)	N1—C4—H4B	108.3
C6—N1—C5	109.41 (12)	C3—C4—H4B	108.3
C7—N1—C5	107.84 (12)	H4A—C4—H4B	107.4
C6—N1—C4	112.76 (11)	N1—C5—H5A	109.5
C7—N1—C4	106.82 (11)	N1—C5—H5B	109.5
C5—N1—C4	111.47 (11)	H5A—C5—H5B	109.5
O2—C1—O1	122.86 (14)	N1—C5—H5C	109.5
O2—C1—C2	124.29 (14)	H5A—C5—H5C	109.5
O1—C1—C2	112.83 (12)	H5B—C5—H5C	109.5
C1—C2—C3	111.95 (11)	N1—C6—H6A	109.5
C1—C2—H2A	109.2	N1—C6—H6B	109.5
C3—C2—H2A	109.2	H6A—C6—H6B	109.5
C1—C2—H2B	109.2	N1—C6—H6C	109.5
C3—C2—H2B	109.2	H6A—C6—H6C	109.5
H2A—C2—H2B	107.9	H6B—C6—H6C	109.5
O3—C3—C4	109.21 (11)	N1—C7—H7A	109.5
O3—C3—C2	111.29 (11)	N1—C7—H7B	109.5
C4—C3—C2	107.87 (11)	H7A—C7—H7B	109.5
O3—C3—H3A	109.5	N1—C7—H7C	109.5
C4—C3—H3A	109.5	H7A—C7—H7C	109.5
C2—C3—H3A	109.5	H7B—C7—H7C	109.5
N1—C4—C3	115.93 (12)		
			
O2—C1—C2—C3	−19.23 (19)	C7—N1—C4—C3	−178.16 (13)
O1—C1—C2—C3	159.77 (12)	C5—N1—C4—C3	−60.59 (16)
C1—C2—C3—O3	70.19 (14)	O3—C3—C4—N1	−78.63 (15)
C1—C2—C3—C4	−170.02 (12)	C2—C3—C4—N1	160.28 (11)
C6—N1—C4—C3	62.93 (16)		

### Hydrogen-bond geometry (Å, º)

**Table:**

*D*—H···*A*	*D*—H	H···*A*	*D*···*A*	*D*—H···*A*
O1—H1···Cl1	0.85 (1)	2.18 (1)	3.022 (1)	176 (2)
O3—H3···Cl1^i^	0.83 (1)	2.51 (2)	3.209 (1)	142 (2)

Symmetry code: (i) −*x*+1/2, −*y*, *z*−1/2.
